# Multicriteria Optimization of Phenolic Compounds Capture from a Sunflower Protein Isolate Production Process by-Product by Adsorption Column and Assessment of Their Antioxidant and Anti-Inflammatory Effects

**DOI:** 10.3390/foods10040760

**Published:** 2021-04-02

**Authors:** Tuong Thi Le, Armelle Ropars, Arnaud Aymes, Jean-Pol Frippiat, Romain Kapel

**Affiliations:** 1 Laboratoire Réactions et Génie des Procédés, Université de Lorraine, Unité Mixte de Recharche CNRS/Ministère (UMR) 7274, LRGP, F-54500 Vandœuvre-lès-Nancy, France; thi-tuong.le@univ-lorraine.fr (T.T.L.); arnaud.aymes@univ-lorraine.fr (A.A.); 2 Stress, Immunity, Pathogens Laboratory, SIMPA, Université de Lorraine, F-54000 Nancy, France; armelle.ropars@univ-lorraine.fr

**Keywords:** chlorogenic acid, response surface methodology, THP-1 macrophage, inflammation, antioxidant

## Abstract

The aim of this study was to valorize liquid effluent from the sunflower protein isolate process by extracting phenolic compounds it contains. To do so, XAD7 resin was used. A multicriteria optimization methodology based on design of experiments showed the optimal conditions were adsorption flow rate of 15 BV/h at pH 2.7, a desorption flow rate at 120 BV/h with ethanol/water 50% (*v*/*v*). The best trade-off between purity and recovery yields resulted in the production of a fraction containing 76.05% of chlorogenic acid (CGA) whose biological properties were evaluated. DPPH and ABTS tests showed that this fraction had a higher radical scavenging capacity than vitamin C. In vitro assays have shown that this fraction, when used at a concentration corresponding to 50 or 100 µM of CGA, does not present any cytotoxicity on human THP-1 cells differentiated into macrophages. In addition, this fraction when added prior to the inflammatory stimulus (LPS) can reduce tumor necrosis factor-alpha (TNF-α) production by 22%, thereby highlighting its protective properties against future inflammation.

## 1. Introduction

Chlorogenic acid (CGA) is a mild polar phenolic compound composed of a quinic acid and a caffeic acid part linked by an ester bond [1,2]. This compound has antioxidant properties throughout free radical scavenging [3,4,5] and metal chelation activities [6,7,8]. Besides, CGA has anti-inflammatory effects [9,10,11,12,13]. According to Drugbank [14], CGA can be used in the pharmaceutical field and a recent report suggests that it could be useful for the preservation of food products [3]. However, the way to use it in the biomedical (human and/or veterinary) field deserves more attention.

CGA can be found in various resources like coffee bean, tea, apple, and sweet potato leaves [5,15,16]. However, these resources are either valorized as commodity or poorly available for CGA production. Sunflower meal (SFM) is also particularly rich in CGA (1.42–4.00% on a dry basis) [17,18,19]. SFM is the oil extraction by-product and is largely available (yearly production about 56 million tons worldwide) [20]. To date, SFM is mainly used for animal nutrition because of its high content in proteins [18,21]. Recently, SFM revealed to be a very interesting source for protein isolate production [18,21,22]. Interestingly, CGA was shown largely predominant in the aqueous by-product yielded by sunflower meal protein isolate production. Such by-products are obtained after saline extraction and a protein purification process either carried out by ultrafiltration or acid precipitation [21,22]. Hence, the capture of CGA from these effluents could offer a very promising valorization way.

Many studies report that the use of mild polar macroporous resins, such as AB-8, NKA-II, and ADS-21 resins, is appropriate for CGA capture from plant extracts with maximum adsorption capacity ranging from 9.83–26.8 mg/g [5,23,24]. Obviously, the adsorption capacity of CGA is favorable under acidic conditions (pH 2–3) because the carboxyl group is protonated which limits CGA polarity and increases the adsorption on mild polar resins [5,24]. Batch adsorption studies allowed to finely understand transport and adsorption phenomena of CGA on resins [23,24]. In brief, CGA transport shows rather low diffusional limitations and the adsorption is mainly governed by physic interactions. Thus, resins should be implemented at temperature around 20 °C [18,25].

However, there is limited information on CGA capture by adsorption column (dynamic adsorption) that are used in industrial applications. Dynamic adsorption is particularly complex since it implies many operating conditions (flow rate, adsorption pH, desorption flow rate, composition of the desorption eluent, etc.) and antagonist performance criteria (purity, recovery, and productivity). This makes it difficult to identify the optimal conditions for the CGA capture. The few dynamic adsorption studies of CGA on macroporous resins showed that the most favorable adsorption pH and flow rate for maximizing dynamic binding capacities or CGA adsorption ratio ranged from 1–3 Bed volume/hour and pH 2–3 respectively with dynamic binding capacity or CGA capture rate around 25 mg/g or 80% [5,21]. The desorption is generally carried out using ethanol–water solvent with ethanol proportion between 40 and 70% (*v*/*v*). Polyphenol purity in the fraction varied in a 15–65% range depending on the material and the ethanol/water ratio [23,24]. The recovery was around 80% [23,24]. Most of these studies were carried out using one factor at a time or design of experiments optimization methods, but were aimed at maximizing a single criterion (most often the dynamic binding capacity). Furthermore, to our knowledge, no study integrates the productivity as a performance criteria, which is crucial for industrial applications. Considering that productivity is antagonist of the binding capacity, the so-called optimized conditions reported in literature are most probably far from the best trade-off.

We previously reported an optimized process for SFM protein isolate production based on protein saline extraction and purification by tangential filtration [22]. In the aqueous by-product, which is the ultrafiltration permeate yielded by the diafiltration step, CGA was shown to be the main phenolic compound [25]. Batch adsorption study revealed that CGA was most favorably captured by XAD7 resin from this by-product. In this study, the effect of adsorption flow rate and pH on dynamic binding capacity, productivity and recovery was investigated by design of experiments (DoE). Then, a multi-objective optimization method of this step was proposed based on the DoE models. Concerning the desorption step, the effect of the ethanol concentration on both CGA purity and recovery was considered. The antioxidant activity of the fraction obtained in the set of optimal conditions was compared to the CGA standard and vitamin C. The anti-inflammatory activity was evaluated using a classical model [26]; the release of the TNF-α pro-inflammatory cytokine by lipopolysaccharide (LPS) treated THP-1 macrophages. These last studies allowed to demonstrate a protective effect of this fraction against a future inflammation. This property could be valorized in the veterinary and human well-being fields and its antioxidants properties in the food industry.

## 2. Materials and Methods

### 2.1. Chemical Reagents

Chlorogenic acid standard (purity ≥ 95%), 1,1-diphenyl-2-picrylhydrazyl (DPPH), 2,2′-azino-bis-(3-ethylbenzothiazoline-6-sulfonic acid) (ABTS), potassium persulfate, ascorbic acid (vitamin C), and macroporous resin XAD7 were purchased from Sigma-Aldrich (St. Louis, MO, USA). High-performance liquid chromatography (HPLC) grade solvents, including acetonitrile, formic acid, and methanol, were purchased from Fisher Chemical (Chapel Hill, NC, USA). Absolute ethanol was from the DASIT group (Heudebouville, France). Sodium chloride was purchased from VWR Chemicals Prolabo (Leuven, Belgium).

### 2.2. Aqueous by-Product from Sunflower Protein Isolate Production

The aqueous effluent used in this study resulted from a sunflower protein isolate process [27]. Briefly, this process was carried out in two steps: firstly, a protein extraction from sunflower cake and secondly, a protein purification by tangential filtration (diafiltration mode). For protein extraction, an appropriate amount of meal and 0.5 M NaCl were mixed at a solid/liquid ratio of 1/9. Then, the pH was adjusted at 7.5 by adding 1 M NaOH. The slurry was agitated (400 rpm) at room temperature during 30 min. The liquid phase was separated from the meal by centrifugation (Thermo Scientific Sorvall LYNX 6000 centrifuge) at 15,000× *g* and 20 °C for 30 min. The aqueous phase was filtered through cellulose filters (Fisherbrand, Waltham, MA, USA). Proteins from the clarified aqueous phase were purified by diafiltration using an Akta Flux 6 ultrafiltration apparatus (GE Healthcare Life Science, Chicago, IL, USA) equipped with a 3 kDa polyether sulfone (PES) membrane with a 4800 cm^2^ surface area (UFP-3-C-6A, GE Healthcare, Chicago, IL, USA). The transmembrane pressure was set at 1.5 bar and the feed rate at 1.5 L/min. The aqueous effluent used in the study was the permeate fraction obtained after flushing the retentate compartment with 6 diavolumes (DV) of 0.5 M NaCl. The generated permeate was acidified to pH 2 by adding 1 M HCl and stored at −20 °C before use.

### 2.3. Column Adsorption

For the adsorption, appropriate volumes of effluent adjusted to pH 2, 3.5, or 5 were injected into columns (16 × 50 mm) packed with XAD7 resin equilibrated with deionized water. The elution was done at 15 BV/h (bed volume/hour), 10 BV/h, or 5 BV/h using an ÄKTA Pure system (GE Healthcare, Sweden). The eluate was fractionated into 10 mL fractions. CGA concentration in each fraction was quantified by HPLC. The elution was monitored by UV detection at 325 nm. The column charge step was stopped when the absorbance at 325 nm corresponded to a CGA concentration corresponding to 10% of its concentration in the feed. Then, the column was washed by 25 BV of deionized water at 120 BV/h and eluted by 99.6% (*v*/*v*) ethanol at 120 BV/h. CGA concentration was also quantified by HPLC in the desorption fraction in order to calculate, for each condition, the three performance criteria associated to the desorption phase (CGA dynamic binding capacity at 10%, i.e., DBC10, process productivity and CGA recovery).

The dynamic binding capacity at 10% (in mg of CGA/g of resin) was calculated as follows:

(1) $$DBC10=\frac{m_{CGA\;ads.}}{m_{resin}},$$

where *m_CGA_*_*ads*._ is the amount of CGA adsorbed onto the resin, and *m_resin_* is the dried resin weight.

The process productivity (in mg of CGA/g of resin/min) was determined as follows:

(2) $$\mathrm{Productivity}\;=\;\frac{m_{CGA\;des.\;}}{m_{resin}\times t\;},$$

where *m_CGA des_*. is the amount of CGA in the desorption fraction (in mg), *m_resin_* is the dried resin weight (in g), and t is the total duration of the adsorption + washing + desorption process.

The recovery (expressed in %) was calculated as follows:

(3) $$\mathrm{Recovery}\;\left(\%\right)=\frac{m_{CGA\;out.}}{m_{CGA\;in.}}100\%,$$

where *m_CGA__in_*. is the mass of CGA introduced at the column inlet determined by HPLC and *m_CGA__out_*. is the amount of CGA in the desorption fraction.

To define the best desorption conditions, the optimal adsorption conditions were applied (15 BV/h at pH 2.7). Then, the column was washed by 25 BV of deionized water and eluted with 30, 50, 70, and 90% of ethanol in water (*v*/*v*) at a flow rate of 120 BV/h as recommended by the manufacturer. The elution was collected every 10 mL and stopped when the UV signal at 325 nm reached the baseline. CGA concentration was determined in each of the 10 mL fractions by HPLC. The ÄKTA Pure system (GE Healthcare, Sweden) performed all dynamic adsorption and desorption steps thanks to the UNICORN software. Fractions containing CGA were pooled and freeze-dried after vacuum concentration for further analysis (CGA and dry matter amounts) and use (antioxidant or anti-inflammatory assays).

CGA purity in the desorption fraction was calculated as follows:

(4) $$\mathrm{Purity}\;\left(\%\right)=\;\frac{m_{CGA\;des.}\;}{\sum^{\text{​}}m_{total\;mass}}100\%,$$

where *m_CGA des_*_._ is the amount of CGA in the desorption fraction and *m_total mass_* is the amount of the dried product.

### 2.4. HPLC Analysis

CGA quantification was carried by size exclusion HPLC as recommended by Sara et al. [22]. The system (Shimadzu Corporation, Kyoto, Japan) was composed of a pump, a degasser (LC-20AD), an auto-sampler (SIL-20AC), a column oven (CTO-20A), and a diode array detector (CPO-M20A). The column used was a Biosep 5 µm SEC-s2000 (300 × 7.8 mm; Phenomenex, Torrance, CA, USA). The mobile phase was composed of formic acid/ultrapure water/acetonitrile (0.1%/55%/45%, *v*/*v*). The temperature was set at 35 °C and the flow rate was 0.6 mL/min. The injection volume was 5 µL. The detection wavelength was 325 nm. Peak identity was confirmed from retention time data with a standard sample to measure CGA concentration and MS analysis. The calibration curve of standard CGA was constructed in a concentration range 0.05–1.25 mg/mL (*y* = 2.51 × 10^7^, *R*^2^ = 0.9983).

### 2.5. Design of Experiments

Design of experiments was used to investigate the influence of adsorption flow rate and pH. The adsorption flow rate (X_1_) was studied in the range of 5 to 15 BV/h. pH was studied in the range of 2 to 5. Considered criteria were DBC10 (in mg/g), productivity (in mg/g/min), and recovery (in %). The face-centered central composite design was generated and analyzed using the MODDE^®^ 7 software form Sartorius Stedim Biotech (Göttingen, Germany). The experimental matrix was composed of 11 combinations of adsorption flow rates (ranging from 5 to 15 BV/h) and pH values (ranging from 2 to 5) including three replications at the central point. The coded setting conditions are presented in Table S1.

The mathematical relationship between factors and responses was described by the following second-degree polynomial equation (Equation (5)):

(5) $$Y=\beta_{0}+\beta_{1}X_{1}+\beta_{2}X_{2}+\beta_{11}X_{1}^{2}+\beta_{22}X_{2}^{2}+\beta_{12}X_{1}X_{2},$$

where *Y* is the response, *β*_0_ is the constant, *β*_1_ and *β*_2_ are the coefficients of linear effects, *β*_11_ and *β*_22_ are the coefficients of quadratic effects, *β*_12_ is the coefficient of interaction effect, *X*_1_ and *X*_2_ are the independent factors.

The obtained models were statistically verified by evaluating coefficient of determination (*R*^2^), residual standard deviation (RSD), and analyze of variance (ANOVA) (regression *p*-value and lack of fit). The significance was considered as statistically significant when *p*-value was <0.05.

### 2.6. Multi-Objective Optimization

Multi-optimization was employed to identify the best conditions for productivity and CGA recovery in terms of adsorption flow rate and pH. The objective function corresponded to the simultaneous maximization of productivity and recovery. The multi-objective problem was solved using the model equations and setting two criteria within the following constraints: maximal productivity and 80% recovery. The multilevel algorithm was built and analyzed using the MATLAB^®^ software from MathWorks (Natick, MA, USA). Generally, the optimization process was divided into several steps. First, an initial population representing a group of individuals (*n* = 2000) was generated. Each individual corresponded to random of dynamic adsorption conditions (adsorption flow rate from 5 to 15 BV/h and pH from 2 to 5) varying by the algorithm. Process performance (dynamic binding capacity, productivity, and recovery) of each individual was calculated on the basis of the model equations. Then, the initial population was evaluated regarding their performances towards fixed criteria. The dominant individuals participated subsequently in the production of a new generation and created solutions were one more time evaluated. Process was repeated until the set of non-dominant solutions was not established.

### 2.7. Antioxidant Activity

#### 2.7.1. DPPH Radical Scavenging Activity

DPPH free radical scavenging activity was studied according to Wu et al. [28] with some modifications. One hundred µL of the samples at 40 µg/mL were mixed with 100 µL of 0.2 mM DPPH solution, prepared in MeOH, into the wells of a microplate. These mixtures were shaken for 30 s and left 30 min in the dark at 25 °C. Then, the absorbance was recorded at 517 nm. The inhibition percentage (%) of radical scavenging capacity was expressed as follows:

(6) $$DPPH\;radical\;scavenging\;\left(\%\right)=\;\frac{\left(A_{DPPH\;-}\;A_{blank}\right)-\left(A_{sample+DPPH}-\;A_{sample+blank}\right)}{A_{DPPH}-A_{Blank}}100\left(\%\right),$$

where *A_DPPH_* is the absorbance of the DPPH solution, *A_blank_* is the absorbance of pure methanol, *A_sample+DPPH_* is the absorbance of DPPH with the sample, and *A_sample+blank_* is the absorbance of pure methanol with the sample.

#### 2.7.2. ABTS Radical Scavenging Activity

ABTS radical scavenging activity was determined using the method described by Re et al. [29] with some modifications. ABTS^+^ was prepared by mixing 7 mM ABTS radical cation stock solution with 2.45 mM potassium persulfate in a 1:1 (*v*/*v*) ratio. This solution was kept in the dark at room temperature for 16 h and thereafter its absorbance at 734 nm was adjusted to 0.70 ± 0.02 by diluting it with a 90% (*v*/*v*) methanol/water. Samples were tested at the following concentrations: 200, 100, 50, 25, and 12.5 µg/mL. Twenty microliters of each sample concentration was mixed with 180 µL of ABTS and incubated 5 min in the dark. Then, the absorbance at 734 nm was read. The following equation was used to calculate the percentage of decrease of the absorbance at 734 nm:

(7) $$ABTS^{.+}\;inhibited\;rate\;\left(\%\right)\;=\;\frac{A_{ABTS}-\;A_{sample+ABTS}}{A_{ABTS}}100\;\left(\%\right),$$

where *A_ABTS_* is the absorbance of ABTS alone and *A_sample+ABTS_* is the absorbance of ABTS in the presence of the sample.

The IC50 value (i.e., the antioxidant concentration showing 50% of maximum antioxidant capacity) was calculated by linear regression of the plot of scavenging activity versus sample concentration. Data are expressed as mean ± standard deviation (S.D), and all experiments were run in triplicate.

### 2.8. Cell Culture and Treatments

The THP-1 human monocytic leukemia cell line was a gift of Dr. E. Emilie (INSERM, Paris, France). THP-1 cells were cultured at 37 °C under 5% CO2 in RPMI 1640 medium supplemented with 10% heat inactivated fetal calf serum, 100 U/mL penicillin, 100 µg/mL streptomycin, 10 mM HEPES, 2 mM l-glutamine, 1 mM sodium pyruvate, and 1x non-essential amino-acids. THP-1 cells, at a density of 0.8 × 106 cells/mL, were differentiated into macrophages with 20 nM of phorbol myristate acetate (PMA) in 24-well plates. After 3 days, differentiated THP-1 cells were incubated for 24 h with 100 ng/mL of LPS added one hour before or after chlorogenic acid standard (CGA) or CGA fraction. Two concentrations of CGA (50 and 100 µM) and two working concentrations of CGA fraction, corresponding to a content of 50 or 100 µM of CGA, were used. All chemicals were purchased from Sigma-Aldrich (St. Louis, MO, USA).

### 2.9. Cell Viability

After 24 h of incubation with LPS and CGA, or LPS and CGA fractions, differentiated THP-1 cell viability was determined using the crystal violet assay. Briefly, cells were washed with phosphate buffer saline (PBS) and incubated with 0.1% crystal violet for 20 min at ambient temperature. Then, cells were carefully washed with PBS and lysed with 10% acetic acid for 20 min. Well contents were homogenized and analyzed by spectrophotometry at 595 nm with a multilabel counter (Wallac-1420, Perkin Elmer, Shelton, CT, USA).

### 2.10. TNF-α Quantification

At the end of the 24 h of incubation with LPS and CGA, or LPS and CGA fraction, cell culture supernatants were harvested in sterile conditions, centrifuged to remove dead cells, and stored at −80 °C until analysis. TNF-α concentrations were determined using the human TNF-alpha Quantikine ELISA kit (R&D Systems, BioTechne Brands, Rennes, France). Assays were performed according to the instructions of the manufacturer, in duplicate, and repeated three to five times. Plates were read at 450 nm with a multilabel counter (Wallac-1420, Perkin Elmer, Shelton, CT, USA).

### 2.11. Data Analysis

For the response surface methodology, using the analysis of variance (ANOVA) given by MODDE 7.0, the statistical parameters, including the determination of coefficients (*R*^2^), regression *p*-value, and lack of fit could be achieved. The mathematical models had criteria *R*^2^ values close to 1. For antioxidant activity, the statistical analysis was performed using Rstudio (version 3.6.1) and *t*-tests. Statistically significant differences were indicated by *p* value < 0.05. For anti-inflammation studies, six independent experiments were performed in triplicates. *t*-tests were used to identify statistically significant differences (*p* ≤ 0.05).

## 3. Results and Discussion

### 3.1. Dynamic Adsorption Step

#### 3.1.1. Effect of pH and Flow Rate on Dynamic Binding Capacity, Recovery, and Process Productivity

In a previous study, the XAD7 macroporous resin was reported to be promising for phenolic compounds capture from an aqueous by-product yielded by a sunflower protein isolate process [25]. In the present study, the dynamic adsorption of the phenolic fraction on XAD7 column from the same by-product was studied using the DoE methodology. The adsorption flow rate and the pH of liquid effluent on the resin dynamic binding capacity (DBC10), process productivity, and recovery were considered. The adsorption flow rate was chosen because it is known to strongly impact dynamic adsorption [21,23]. The effect of the pH was considered because it was reported to impact the adsorption of main sunflower phenolic compounds (i.e., chlorogenic acid, CGA) on macroporous resins [5,15,21,24,30]. Criteria like DBC and recovery are often included in column adsorption studies but process productivity is more rarely took into consideration whereas it is crucial for industrial applications. The chosen pH ranges from 2 to 5 because above pH 5, CGA is known to be converted into its oxidized form [27]. The flow rate varied from 5 to 15 BV/h as recommended by the supplier. The results related to polyphenol amounts were expressed in CGA mass because this phenolic compound constitutes 1.13 ± 0.21% of the liquid by-product used in this study [25].

The obtained mathematical models are shown in Equations (8)–(10). Regression parameters (unscaled and scaled), and statistical verification of model performances using the ANOVA test are presented in Table 1.

**Figure 1:**
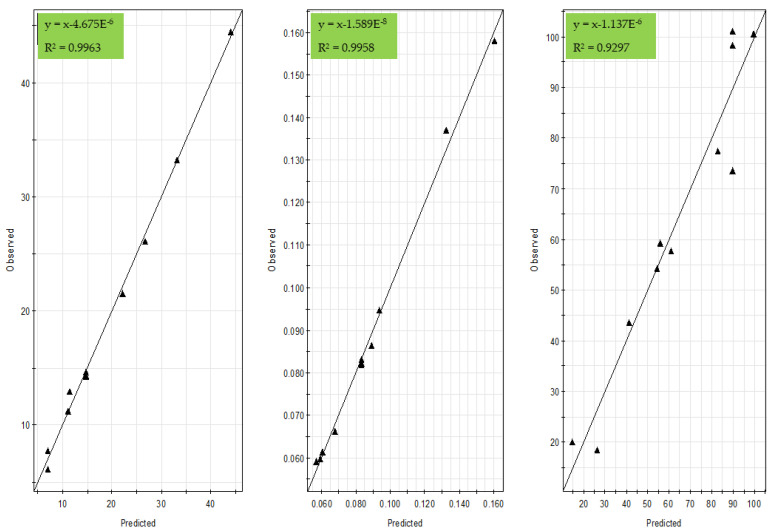
Comparison of model-predicted and actual values for dynamic binding capacity (**left**), productivity (**middle**), and recovery (**right**) responses.

The mathematical models were characterized by high indexes of regression coefficient between predicted and observed values (*R*^2^ = 0.996 for dynamic binding capacity at 10% (DBC10), *R*^2^ = 0.996 for productivity, and *R*^2^ = 0.93 for recovery) (Figure 1).

These models were also observed with low regression *p*-values (0.00 for dynamic binding capacity, 0.00 for productivity, and 0.007 for recovery) and insignificant lack of fit (0.28 for dynamic binding capacity, 0.22 for productivity, and 0.877 for recovery). The calculated and experimental plots are presented in Figure 1. These data suggested a high fitted and reliability of models for the prediction of the assessed process:

(8) $$Y_{1\left(dynamic\;binding\;capacity\;at\;10\%,\;DBC10\right)}=109.19-4.31X_{1}-29.89X_{2}+0.085X_{1}^{2}+\;2.39X_{2}^{2}-0.44X_{1}X_{2},$$

(9) $$Y_{1\left(productivity\right)}=0.10+0.018X_{1}-0.05X_{2}-0.0003X_{1}^{2}+\;0.0075X_{2}^{2}-0.0023X_{1}X_{2},$$

(10) $$Y_{2\left(recovery\right)}=-139.43-0.68X_{1}+147.49X_{2}+0.067X_{1}^{2}-\;21.53X_{2}^{2}-0.67X_{1}X_{2},$$

where *X*_1_ is the adsorption flow rate (BV/h), and *X*_2_ is the pH of the permeate solution.

**Figure 2:**
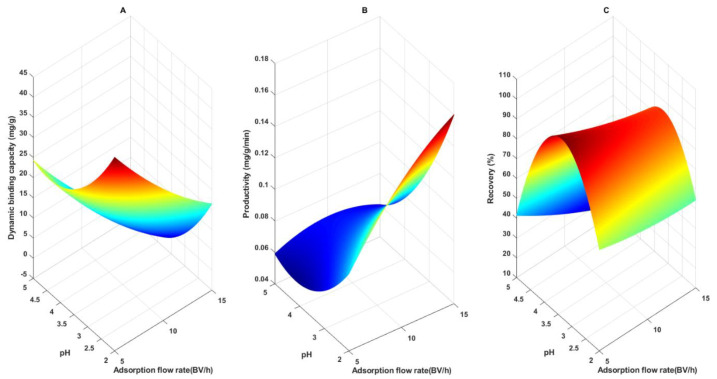
3-dimensional plots presenting the effects of adsorption flow rate and pH on chlorogenic acid (CGA) yield. pH versus adsorption flow rate on adsorption binding capacity (**A**), productivity (**B**), and recovery (**C**).

Figure 2A shows the effect of pH and flow rate on DBC10. At pH 2, DBC10 increases from 26.1 to 44.47 mg/g when flow rate decreases from 15 to 5 BV/h. This trend is observed whatever the pH value. This behavior is classically explained by reduced diffusional limitations inside resin pores at low flow rate. It has been observed in many reports regarding CGA adsorption onto macroporous resins. Hence, CGA adsorption from *Helianthus tuberosus* L. leaves extract on ADS-21, from potato leaves on AB-8, from *Eupatorium adenophorum* Spreng extract onto NKA-II were recommended to range from 1 to 3 BV/h [5,23,24]. However, it can be noticed that in this case, DBC10 is only increased by 41.5% at pH 2 when the flow rate is decreased from 15 to 5 BV/h while others observed an 80% increase when flow rate was decreased from 7 to 3 BV/h [21]. Furthermore, DBC10 at pH 5 and 5 BV/h revealed close (70%) to maximal CGA binding capacity [25]. Interestingly, kinetic study of batch CGA adsorption on XAD7 revealed that a strong intra-pore diffusional limitation was observed for only 10–15% of the total resin area. This could explain the mild effect of the flow rate on CGA DBC10 on XAD7.

A pH decrease clearly had a positive effect on DBC10 (increase from 11.28 mg/g to 44.47 mg/g when decreasing pH from 5 to 2 at 5 BV/h). This is consistent with other CGA adsorption studies from various sources (sweet potato leaves and sunflower meal) on other macroporous resins (XAD16, AB-8) [5,21] Le et al. [25]. This can be explained by a strong reduction of polarity at pH < 4 due to the ionization of the carboxyl group of CGA quinic acid part (pK_a_ = 3.95). This polarity reduction would improve the association constant with mild apolar resins like XAD7. Hence, most of the authors recommended to perform CGA adsorption at pH 2.

Figure 2B shows the impact of pH and flow rate on process productivity. The positive effect of pH decrease on productivity was expected. It results from the above observed increase in CGA affinity for XAD7. Most interestingly, the productivity was increased by 45.57% (from 0.086 to 0.158 mg/g/min) when the flow rate was increased from 5 to 15 BV/h. It indicates that highest adsorption velocity at high flow rate largely compensates lower binding capacity. To our knowledge, this has never been observed yet. It clearly shows that high flow rate should be recommended contrarily to other reports [5,21,23,24].

CGA recovery is a crucial performance criterion for industrial applications. In this study, recovery represents the ratio of desorbed CGA after the adsorption process (using 100% ethanol) over the amount of injected CGA. Hence, this term lumps both CGA desorption yield and CGA loss during column washing prior to description. Figure 2C shows the effect of adsorption pH and flow rate on recovery. Above pH 3.5, a positive effect of decreasing pH and flow rate was observed as with DBC10. Below pH 3.5, a strong negative effect of the pH with no or few effect of the flow rate can be noticed. To our knowledge, such a parabolic effect of adsorption pH on the recovery of a phenolic compound after desorption on macroporous resin has never been observed. The effect of the flow rate above pH 3.5 was also surprising because this condition should only impact criteria related to the adsorption.

As mentioned above, the effect of the pH and flow rate on CGA recovery seems strongly related to its dynamic adsorption. At flow rate and pH ranging from 10 to 15 BV/h and 3.5 to 5 respectively, DBC10 remained rather low (between 6.13 and 14.67 mg/g, but mostly around 10 mg/g while the maximum DBC10 value was higher than 40 mg/g). Hence, in this range of poor CGA adsorption, the CGA content in the mobile phase at the end of the adsorption step that was flushed by the washing step was not negligible and impacted the overall recovery. This probably explains the observed effect on CGA recovery. Obviously, it can be assumed that this bias had a poor impact at DBC10 values above 20 mg/g that were reached under pH 4.

The negative effect of pH under 3.5 was more puzzling. As explained above, in this pH range, the DBC10 value was high enough for neglecting CGA loss during the washing step. Hence, the recovery was only impacted by CGA desorption yield. We suggested that the increased CGA adsorption capacity in this pH condition was due to a predominance of its deionized form. We can therefore further hypothesize that the deionized form needs a less polar solvent than ethanol for full desorption.

In any cases, previous studies suggested to implement CGA adsorption at low pH (around 2) and adsorption flow rate (less than 5 BV/h) [5,23,24]. Our results indicate that this should be reassessed. We therefore used the mathematical models of the DoE regression tools to carry out a multicriteria optimization of the process.

#### 3.1.2. Multi-Objective Optimization

Multi-objective optimization was used to identify optimal conditions of the adsorption process, i.e., that give the maximum yield of CGA. The best conditions were identified based on the highest productivity at a fixed recovery of 80%. These studies indicated that the optimum is reached with an adsorption flow rate of 15 BV/h at pH 2.7. Experimental results were then compared to predicted values given by modeling (Table 2). Experimentally-determined productivity and recovery were 0.128 ± 0.19 mg/g/min and 78.77 ± 3.61%, respectively. Predicted values were quite comparable to observed values as they were within the 95% confidence level. These results indicate that the regression models appropriately fitted experimental data. Therefore, the adsorption flow rate of 15 BV/h and the pH value of 2.7 were chosen as optimal for the dynamic adsorption process.

#### 3.1.3. Dynamic Desorption Step

The last criterion to integrate for optimization of adsorption is the purity of the fraction. This criterion is known to depend essentially on desorption conditions (i.e., the desorption solvent composition). Therefore, an appropriate solvent for CGA desorption had to be evaluated and selected after optimization of dynamic adsorptions. Ethanol has the advantages of being low-cost and eco-friendly. It was therefore used as eluent in this study. During the desorption process, ethanol concentration varied from 30% to 90%. The desorption flow rate was fixed at 120 BV/h as recommended by the manufacturer.

Table 3 shows that the highest purity of CGA was obtained when elution was performed using EtOH 50% (*v*/*v*) (76.05 ± 0.00%), followed by EtOH 70% (*v*/*v*) (71.89 ± 0.07%) and EtOH 90% (*v*/*v*) (72.31 ± 1.21%). The highest recovery was reached with EtOH 90% (*v*/*v*) (74.22 ± 0.95%), followed by EtOH 70% (*v*/*v*) (71.77 ± 1.71%), and EtOH 50% (*v*/*v*) (71.38 ± 1.59%). Lowest purity and recovery were noted with EtOH 30% (*v*/*v*). As suggested by Sun et al. [24], it is likely that CGA is not fully desorbed by low ethanol concentration like 30% (*v*/*v*) contrarily to other minor polar impurities. The best compromise between purity and recovery was observed with EtOH 50% (*v*/*v*) (76.05 ± 0.004%). In this set of conditions, the purity was better than reported by Sun et al. [24] and Liu et al. [23] (65.2% and 22.17% respectively at 60% and 40% (*v*/*v*) ethanol). CGA overall recovery was slightly lower though (71.38 ± 1.59%). Weisz et al. [18] also showed slightly higher recovery (84.3%) using 50% (*v*/*v*) 2-propanol elution. But these three studies reported flow rates that are far from those recommended by the manufacturers for industrial applications and used in this study (i.e., 2BV/h vs. 15BV/h). This most probably explains the observed discrepancies.

**Figure 3:**
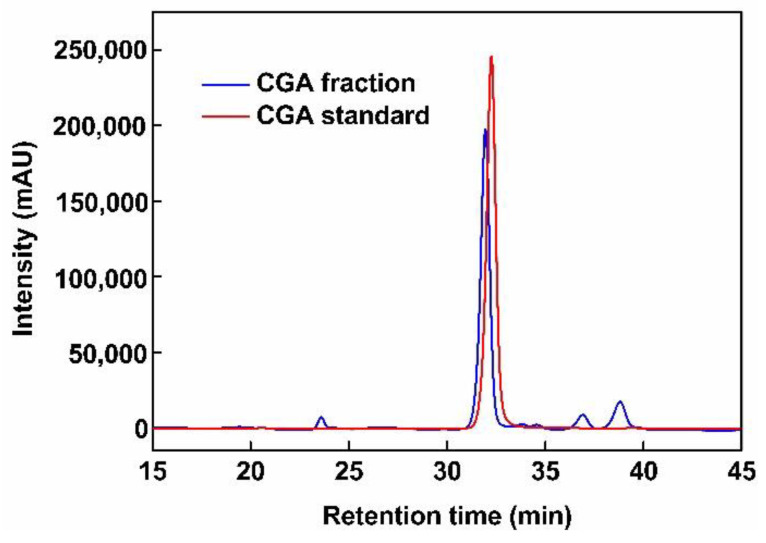
HPLC chromatogram of the sample after passing through the XAD7 resin column (fraction), and of pure chlorogenic acid (standard).

Figure 3 shows the size exclusion HPLC chromatogram at 325 nm of standard CGA (5-CQA) and of the desorbed fraction obtained at 50% (*v*/*v*) ethanol. Standard CGA shows a single peak at 32 min of elution time analyzed by mass spectrometry as CGA (5-CQA). The fraction shows a main peak at the elution time and two minor peaks at 37 and 38.5 min of elution times identified by MS as 3- and 4-caffeoylquinic acid (3- and 4-CQA) i.e., the two other CGA isomers. Traces of caffeic acid were also detected at 23 min. Hence, the largest part of the UV signal at 325 nm (80.69%) was CGA and 5-CQA was the major component (76.05%). To our knowledge, such characterization of CGA fraction has never been reported. Obviously, CGA fraction differed from the standard and is composed of unidentified minor components that could interfere with its reported bioactivity. Hence, the antioxidant effect of the fraction was compared to CGA standard reference and vitamin C.

### 3.2. In Vitro Antioxidant Activity

**Figure 4:**
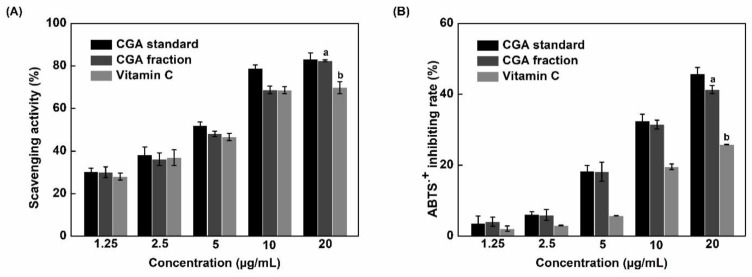
Scavenging activity of the CGA fraction compared to pure CGA (standard) and vitamin C determined using DPPH (**A**) and ABTS (**B**) assays. Bars labeled with different letters are significantly different (*p* < 0.05).

To evaluate the scavenging potential of the CGA fraction by comparison to pure CGA and vitamin C at the same concentrations, DPPH and ABTS assays were conducted [31,32]. As shown in Figure 4, the scavenging potential of all samples gradually increased with concentration, regardless of the method used. At the highest concentration (20 µg/mL), CGA fraction and pure CGA had the same scavenging activity, as revealed by the DPPH assay (83.01 ± 0.16% and 82.36 ± 0.38%), and their antioxidant activity was 1.2 times higher than vitamin C. Besides, Figure 4B shows that the ABTS^+^ inhibition rate of the CGA fraction, pure CGA, and vitamin C at 20 µg/mL were 41.32 ± 0.12, 45.81 ± 0.06, and 25.85 ± 0.13%, respectively. The antioxidant activity of vitamin C, as determined using the ABTS assay, was weaker than that of the CGA fraction and pure CGA (1.60–1.78 times lower). Furthermore, as shown in Table 4, very interestingly, vitamin C IC50 values were always higher than those obtained using the CGA fraction or pure CGA, thereby showing that the order of antioxidant capacity was: pure CGA ≥ CGA fraction > vitamin C (*p* < 0.05). The antioxidant properties of our fraction is therefore much higher than the one of vitamin C which is frequently used as reference in the literature.

**Figure 5:**
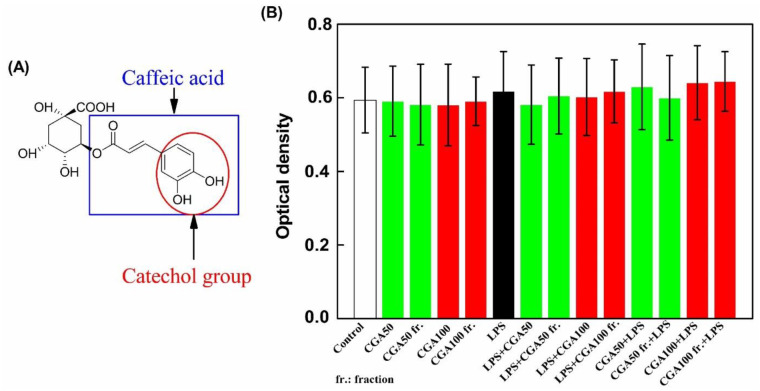
Chlorogenic acid (CGA) structure (**A**) and effects of this pure compound or of the CGA fraction on the viability of differentiated THP-1 cells (**B**). Two concentrations (50 and 100 µM) of pure chlorogenic acid used as references and two working concentrations of the fraction corresponding to a content of 50 or 100 µM of CGA (CGA50 fr. and CGA100 fr.) were used. Differentiated THP-1 cells were incubated for 24 h either with these products alone or with 100 ng/mL of lipopolysachharide (LPS) added one hour before or after the addition of pure CGA or CGA fraction. Then, cell viability was analyzed by crystal violet assay and optical density (O.D.) was measured at 595 nm. Data are presented as mean ± S.D. This figure is representative of six independent experiments realized in triplicates. “Control” corresponds to a culture in which no product was added. “LPS + CGA50” indicates that LPS was added before CGA at the concentration of 50 µM while “CGA50 + LPS” indicates that CGA at the concentration of 50 µM was added before LPS.

As shown in Table 3, the fraction used in antioxidant tests contained 76% of CGA. In both assays, this fraction and pure CGA showed a similar antioxidant activity. This observation indicates that CGA strongly contributed to radical scavenging activity in the CGA fraction. This activity is related to structure characteristics such as the number of hydroxyl groups (-OHs), and electron-donating activity [33]. As shown in Figure 5A, CGA is formed by an ester bond between caffeic acid and quinic acid. According to Natella et al. [34], the number of hydroxyl groups on phenolic acids contributes as positive moieties to their antioxidant effects. These authors found that the antioxidant activity depends on the number of hydroxyl groups with the following priority: tri-hydroxyl phenolic acids > di-hydroxyl phenolic acids (catechol group) > mono-hydroxyl phenolic acids. The presence of two hydroxyl groups (catechol group) in the caffeic acid part of CGA agrees with this rule and contributes to explain its strong antioxidant activity.

Given the observed antioxidant properties of the CGA fraction and its eco-friendly production process, it can be considered as an interesting natural antioxidant for the food industry.

### 3.3. Cytotoxicity and Anti-Inflammatory Activity of the CGA Fraction

THP-1 cells differentiated into macrophages were used to analyze the effects of the CGA fraction on cell viability and LPS-induced pro-inflammatory response. Chlorogenic acid was used as reference in these experiments because it is the major compound found in this fraction (see Table 3). Two conditions were used to mimic two different cellular states. First condition consisted of pre-incubating differentiated THP-1 cells with 100 ng/mL of LPS to induce a pro-inflammatory state and, one hour later, to add the CGA fraction at working concentrations corresponding to 50 or 100 µM of CGA. The purpose of this approach was to determine if this fraction was able to counter the LPS-induced pro-inflammatory response. The second condition was the opposite, it consisted of incubating THP-1 cells with the CGA fraction, to potentially promote an anti-inflammatory cellular state, and one hour later to add 100 ng/mL of LPS. In both cases, cell viability and TNF-α production, a major pro-inflammatory cytokine, were analyzed after 24 h of treatment.

#### 3.3.1. Cytotoxicity

As shown in Figure 5B, whatever the treatment, no alteration of cell viability was detected using the crystal violet assay. Pure CGA and the CGA fraction, at the two tested concentrations, had no impact on cell viability (cell viability >97%). Thus, impurities remaining in the fraction did not affect cell viability. Consequently, these experimental settings and concentrations were used to evaluate the production of TNF-α by THP-1 macrophages.

#### 3.3.2. Anti-Inflammatory Activity of the CGA Fraction

**Figure 6:**
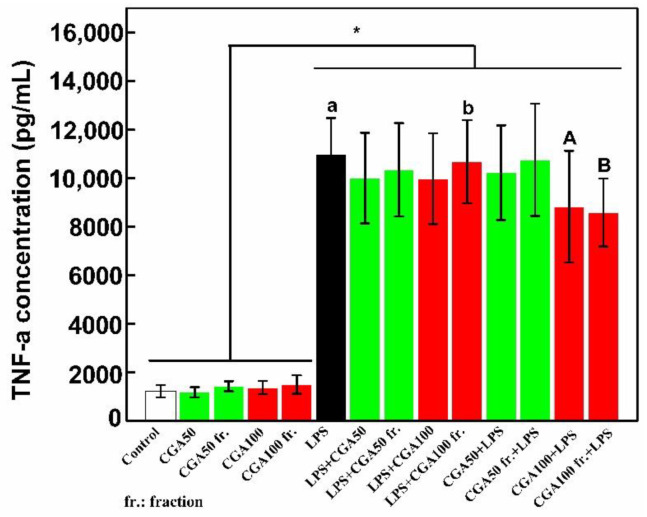
When added before LPS, CGA and the CGA fraction reduce the production of TNF-α by differentiated THP-1 cells. Two concentrations (50 and 100 µM) of pure CGA, used as references, and two working concentrations of the CGA fraction corresponding to a content of 50 or 100 µM of CGA (CGA50 fr. and CGA100 fr.) were used. Differentiated THP-1 cells were incubated for 24 h either with these products alone or with 100 ng/mL of LPS added one hour before or after the addition of pure CGA or the CGA fraction. Cell culture supernatants were then collected and used to quantify TNF-α production by ELISA. This figure is representative of six independent experiments performed in triplicates. Data are presented as mean ± S.D. *t*-tests were used to identify statistically significant differences. Asterisk indicates a significant difference between samples with and without LPS treatment groups (* *p* < 0.05). Bars labeled with the different lowercase letters and uppercase letters are significantly different (*p* < 0.05). “Control” corresponds to a culture in which no product was added. “LPS + CGA50” indicates that LPS was added before CGA at the concentration of 50 µM while “CGA50 + LPS” indicates that CGA at the concentration of 50 µM was added before LPS.

As shown in Figure 6, pure CGA and the CGA fraction had no pro-inflammatory effects when added alone on THP-1 cells differentiated in macrophages. The fact that the CGA fraction is neither cytotoxic nor pro-inflammatory on its own is interesting in term of future potential biomedical valorization. When LPS was added, as expected, a strong increase of TNF-α secretion was observed (indicated by “a” in Figure 6). Furthermore, and very interestingly, we noted that when pretreated with CGA or the CGA fraction at 100 µM, cells produced less TNF-α in response to LPS by comparison to cells receiving only LPS (statistically significant reductions of 20 and 22%, respectively indicated by “c” and “d” in Figure 6). This means that pure CGA and the CGA fraction at 100 µM are able to induce an anti-inflammatory state in THP-1 cells differentiated in macrophages and that impurities remaining in the CGA fraction did not affect this capacity. This effect was not observed when pure CGA or the CGA fraction at 100 µM was added one hour after LPS indicating that if the pro-inflammatory process is initiated, CGA or the CGA fraction is unable to counter this phenomenon.

These results show that a fraction obtained from a low value industrial liquid effluent possesses preventive functions against inflammation, thereby potentially allowing its valorization as a food complement in the biomedical (human and/or veterinary) field. Indeed, intensive breeding is a source of stress leading to an increase of inflammation that affects health and well-being thereby impacting products quality [35,36]. Acute and chronic stress, two characteristics of our actual life style, also induce inflammation [37]. Thus, the properties of this fraction could be of interest for humans [38,39]. Indeed, macrophages are part of the first line of defense against infection and/or tissue injury, and are key actors of the inflammatory process through the production of various mediators such as the pro-inflammatory cytokine TNF-α. Thus, phenolic compounds reducing TNF-α production are promising agents to moderate inflammation. Of course, this effect will have to be confirmed in in vivo studies using stress murine models.

## 4. Conclusions

This study presents an effective way to separate and purify CGA from an industrial liquid by-product resulting from a sunflower protein isolate process. Optimal conditions, based on the response surface methodology for the enrichment of phenolic compounds from sunflower meal, were defined as follows using the XAD7 resin: adsorption flow rate of 15 BV/h, pH 2.7, desorption with EtOH 50% (*v*/*v*). These conditions successfully generated enough product, with a purity of 76.05 ± 0.00% and without using toxic solvents, to evaluate its antioxidant and anti-inflammation properties. The DPPH and ABTS assays showed that the obtained fraction was a more powerful radical scavenger than vitamin C. Furthermore, this fraction showed no cytotoxicity on a human macrophage cell line and reduced LPS-induced TNF-α production by 22%. We therefore propose valorizing this abundant effluent to produce a natural phenolic compound, CGA, which possesses antioxidant and anti-inflammatory properties, but no cytotoxic effects.

## Figures and Tables

**Table 1:** Regression coefficients of the predicted polynomial models for productivity and recovery.

Regression Coefficient			Response	
		Y_1_ (Dynamic Binding Capacity, mg/g)	Y_2_ (Productivity, mg/g/min)	Y_3_ (Recovery, %)
Unscaled	β_o_^a^	109.19	0.10	−139.43
	β_1_^b^	−4.31	0.018	−0.68
	β_2_^c^	−29.89	−0.05	147.49
	β_11_^d^	0.085	−0.0003	0.07
	β_22_^e^	2.39	0.0075	−21.53
	β_12_^f^	0.44	−0.0023	−0.67
Scaled and centered	β_o_	14.71	0.083	89.56
	β_1_	−5.35	0.018	−8.38
	β_2_	−13.11	−0.032	−14.85
	β_11_	2.12	−0.0080	1.67
	β_22_	5.38	0.017	−48.44
	β_12_	3.30	−0.018	−5.003
Statistic model parameter				
*R* ^2^		0.996	0.996	0.93
RSD ^g^		1.005	0.003	11.09
Regression *p*-value		0.00	0.00	0.007
Lack of fit		0.28	0.22	0.877

^a^ constant; ^b^ coefficient of the linear effect of adsorption flow rate; ^c^ coefficient of the linear effect of pH; ^d^ coefficient of the quadratic effect of adsorption flow rate; ^e^ coefficient of the quadratic effect of pH; ^f^ interaction coefficient of adsorption flow rate and pH; ^g^ residual standard deviation.

**Table 2:** Comparison between the predicted and experimental values in the dynamic adsorption process.

Condition	Response Value	
	Productivity (mg/g/min)	Recovery (%)
Predicted values	0.125	79.66
Experimental values	0.128 ± 0.19	78.77 ± 3.61

**Table 3:** Purity and recovery of chlorogenic acid with different ethanol concentrations.

EtOH Concentration (%)	Purity (%)	Recovery (ads. + des.) (%)
30	53.29 ± 0.12	60.05 ± 0.32
50	76.05 ± 0.00	71.38 ± 1.59
70	71.89 ± 0.07	71.77 ± 1.71
90	72.31 ± 1.21	± 0.95

**Table 4:** IC50 values deduced from the DPPH and ABTS assays.

Compound	IC50/DPPH (µg/mL)	IC50/ABTS (µg/mL)
Pure CGA (standard)	5.76 ± 0.02a	20.38 ± 0.02a
CGA fraction	7.05 ± 0.01b	22.52 ± 0.03b
Vitamin C	7.26 ± 0.02c	36.31 ± 0.01c

Different letters in the same column for the IC50/DPPH and IC50/ABTS indicate significant differences (*p* < 0.05) between individual sample treatments.

## Data Availability

The data presented in this study are available in this published article.

## Supplementary Materials

The following are available online at https://www.mdpi.com/article/10.3390/foods10040760/s1, Table S1: Coded factors and experimental design levels for responses surface.

## Abbreviations

CGA	Chlorogenic acid
3-CQA	3-Caffeoylquinic acid
4-CQA	4-Caffeoylquinic acid
5-CQA	5-Caffeoylquinic acid
MS	Mass spectrometry
SFM	Sunflower meal
HPLC	High-performance liquid chromatography
DBC	Dynamic binding capacity
DBC10	Dynamic binding capacity at 10%
DoE	Design of Experiment
BV	Bed volume
LPS	Lipopolysaccharide
TNF-α	Tumor necrosis factor-alpha
DV	Diavolume
DPPH	1,1-diphenyl-2-picrylhydrazyl
ABTS	2,2′-azino-bis-(3-ethylbenzothiazoline-6-sulfonic acid)
IC50	Inhibitory concentration at 50% of scavenging rate
RSD	Residual standard deviation
ANOVA	Analyze of variance
SD	Standard deviation
PMA	Phorbol myristate acetate
ELISA	Enzyme-Linked Immunosorbent Assay
PBS	Phosphate buffer saline
BHA	Butyl hydroxyl anisol
BHT	Butylated hydroxyl toluene
OD	Optical density
Des.	Desorption
Ads.	Adsorption
UV	Ultraviolet
*v*/*v*	Volume/volume
rpm	Revolution per minute
