# Supplementary material for: Multicriteria Optimization of Phenolic Compounds Capture from a Sunflower Protein Isolate Production Process by-Product by Adsorption Column and Assessment of Their Antioxidant and Anti-Inflammatory Effects
Source: Foods. 2021 Apr 2;10(4):760. doi: 10.3390/foods10040760 (PMC8066219; doi:10.3390/foods10040760)
Supplement: Supplementary file 1 [file foods-10-00760-s001.pdf]

foods

Article

Multicriteria Optimization of Phenolic Compounds Capture from a Sunflower Protein Isolate Production Process by-Product by Adsorption Column and Assessment of Their Antioxidant and Anti-Inflammatory Effects

Tuong Thi Le [1,2], Armelle Ropars [2], Arnaud Aymes [1], Jean-Pol Frippiat [2,*,†] and Romain Kapel [1,*,†]

1 Laboratoire Réactions et Génie des Procédés, Université de Lorraine, Unité Mixte de Recharche CNRS/Ministère (UMR) 7274, LRGP, F-54500 Vandœuvre-lès-Nancy, France; thi-tuong.le@univ-lorraine.fr (T.T.L.); arnaud.aymes@univ-lorraine.fr (A.A.)
2 Stress, Immunity, Pathogens Laboratory, SIMPA, Université de Lorraine, F-54000 Nancy, France ; thi-tuong.le@univ-lorraine.fr (T.T.L.); armelle.ropars@univ-lorraine.fr (A.R.)
* Correspondence: jean-pol.frippiat@univ-lorraine.fr (J.-P.F.); romain.kapel@univ-lorraine.fr (R.K.)
† Co-supervised this study.

Supplementary Materials: The following are available online at www.mdpi.com/xxx/s1, Table S1: Coded factors and experimental design levels for responses surface.

Table 1. Coded factors and experimental design levels for responses surface.

Factor	Unit	Coded Symbol	Coded Variable Levels		
			−1	0	1
Adsorption flow rate	BV/ h	X_1	5	10	15
pH		X_2	2	3.5	5
